# Computational workflow for functional characterization of COVID-19 through secondary data analysis

**DOI:** 10.1016/j.xpro.2021.100873

**Published:** 2021-09-24

**Authors:** Sudhir Ghandikota, Mihika Sharma, Anil G. Jegga

**Affiliations:** 1 Division of Biomedical Informatics, Cincinnati Children’s Hospital Medical Center, Cincinnati, OH, USA; 2 Department of Computer Science, University of Cincinnati College of Engineering, Cincinnati, OH, USA; 3 Department of Pediatrics, University of Cincinnati College of Medicine, Cincinnati, OH, USA

**Keywords:** Bioinformatics, Single Cell, Health Sciences, Genomics, RNAseq, Immunology, Gene Expression, Systems biology

## Abstract

Standard transcriptomic analyses cannot fully capture the molecular mechanisms underlying disease pathophysiology and outcomes. We present a computational heterogeneous data integration and mining protocol that combines transcriptional signatures from multiple model systems, protein-protein interactions, single-cell RNA-seq markers, and phenotype-genotype associations to identify functional feature complexes. These feature modules represent a higher order multifeatured machines collectively working toward common pathophysiological goals. We apply this protocol for functional characterization of COVID-19, but it could be applied to many other diseases.

For complete details on the use and execution of this protocol, please refer to Ghandikota et al. (2021).

## Highlights

• Steps for meta-analysis of multiple transcriptomic studies and protein interactions
• Network analysis-based workflow to identify gene and functional modules
• Data-driven higher-order functional features provide a basis for characterizing disease

## Graphical abstract

**Figure:**
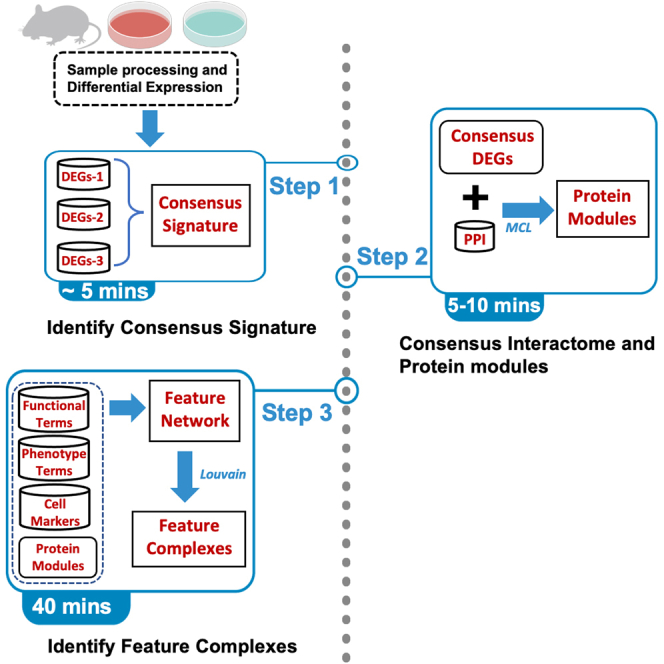

## Before you begin

### Transcriptomic study selection

1. Transcriptomic studies associated with a given disease or phenotype need to be selected. At least two gene expression studies are required in our meta-analysis approach for identifying consensus DEGs.
2. We used the NCBI Gene Expression Omnibus (GEO) repository (Barrett et al., 2013) (https://www.ncbi.nlm.nih.gov/geo/info/). Conversely, other public repositories such as the ArrayExpress (Athar et al., 2019) can be used in this step.
3. In the SARS-CoV-2 case study, we used transcriptomic data from two *in vitro* (Calu-3 and Vero E6 cells) models and one *in vivo* (Ad5-hACE2-sensitized mice) model of SARS-CoV-2 infection. The Calu-3 model (GSE147507) (Blanco-Melo et al., 2020) is based on six samples with three each of SARS-CoV-2 infected or mock treated samples. The second *in vitro* model is based on mRNA profiles of 24h post-SARS-CoV-2-infection (USA-WA1/2020, multiplicity of infection = 0.3) in Vero E6 cells (kidney epithelial cells extracted from an African green monkey (GEO: GSE153940)) (Riva et al., 2020). The third transcriptomic dataset is from a mouse model of Ad5-hACE2-sensitized mice (GSE150847) (Sun et al., 2020) that develop pneumonia after infection with SARS-CoV-2.

***Optional:*** An optional step is to use disease-specific protein-protein interaction (PPI) data. In our SARS-CoV-2 protocol, we used the SARS-CoV-2-human virus-host interactome data (Gordon et al., 2020), consisting of 332 proteins, to construct an integrated virus-host interactome.

### Human lung cell markers and genome-wide associations

4. Besides the transcriptomic signature and the optional PPI data, our meta-analysis approach also uses several non-disease data elements that can be considered based on the type of disease/phenotype investigated.
5. In this protocol, for characterizing SARS-CoV-2 infection, we use single-cell RNA-seq (scRNA-seq) markers from three human lung studies (Adams et al., 2020; Habermann et al., 2020; Travaglini et al., 2020) and genome-wide association study (GWAS) data from both PheGenI (Ramos et al., 2014) and GWAS Catalog (Buniello et al., 2019).
6. In case of lung single cell markers, only significant gene markers (FDR p-value ≤ 0.05; logFC ≥ 0.5) are used for enrichment analysis. On the other hand, GWA trait associations are limited to vulnerability loci with an association p-value ≤10^-5^.

### Sample processing

7. After selecting the relevant gene expression data sets, raw data needs to be downloaded and processed to generate raw counts and normalized transcript per million (TPM) for all samples. Our SARS-CoV-2 protocol used the CSBB-v3.0 toolkit for both sample processing and DE analysis as shown below:

perl CSBB-v3.0_MacOS.pl (or) CSBB-v3.0_Linux.pl ProcessPublicData

 Path_to_SRA-DATA_Table

 Path_to_Folder_to_write_results

8. Instructions for installing the toolkit are available on the project GitHub page at https://github.com/praneet1988/Computational-Suite-For-Bioinformaticians-and-Biologists. The .sra files associated with each individual sample are downloaded using the Prefetch utility of the NCBI SRA Toolkit (https://github.com/ncbi/sra-tools). To download all the samples simultaneously, an SRA data table is required and can be created based on the format shown here https://github.com/praneet1988/Computational-Suite-For-Bioinformaticians-and-Biologists/blob/master/Test_Files/SRA_DATA_TABLE.txt.
9. Next, the .sra files are converted to FASTQ format using fastq-dump utility of the SRA toolkit. The final step involves mapping to a transcriptome assembly of interest using bowtie2 (Langmead and Salzberg, 2012) and quantifying the mapped reads using the RSEM package (Li and Dewey, 2011). Alternatively, other methods like kallisto (Pseudo-aligner) (Bray et al., 2016) and Salmon ( Pseudo-aligner) (Patro et al., 2017) can be used for quantifying transcript abundance. Similarly, for the transcript mapping step, other aligner softwares such as STAR (Dobin et al., 2013) can be used.
10. In this protocol, we implemented the above sample processing steps to generate raw sample counts in human Calu-3 model (GSE147507). For the other two studies, we downloaded the raw counts provided by the authors in the respective GEO repositories. The following path in our protocol GitHub repository https://github.com/SudhirGhandikota/COVID19_secondary_analysis/tree/main/input_data/Count%20Data contains the raw counts from all the three studies. These raw counts can then directly be used in the DE analysis step (explained below).

### Differential expression analysis

11. The final pre-processing step involves performing differential expression (DE) analysis, individually in each of the transcriptomic datasets. The following script can used in this step:
  perl CSBB-v3.0_MacOS.pl (or) CSBB-v3.0_Linux.pl
   DifferentialExpression
   Path_To_Counts_File No_of_Control_Samples
   No_of_Treatment_Samples
   Counts_Threshold_Filtering No_of_samples_to_filter_per_Gene
   Normalization_Type
  Command-line parameters accepted for the DE module are:
  a. Path_To_Counts_File: Raw counts file from the previous step.
  b. No_of_Control_Samples and No_of_Treatment_Samples: Number of controls and treatment samples respectively.
  c. Counts_Threshold_Filtering: Minimum raw count threshold (for filtering samples).
  d. No_of_samples_to_filter_per_Gene: Minimum number of samples in which a gene must be expressed (for filtering genes). In our SARS-CoV-2-specific implementations, we have set Counts_Threshold = 10 and No_of_samples = 3.
  e. Normalization_Type: Type of normalization (“UpperQuantile” or “UpperQuantile+Empirical”).
  f. Further descriptions about these parameters can be found at https://github.com/praneet1988/Computational-Suite-For-Bioinformaticians-and-Biologists.

12. Prior to DE analysis, upper-quartile normalization (Bullard et al., 2010) is applied on the raw RNA-seq counts by using EDASeq (Risso et al., 2011) R package (http://bioconductor.org/packages/release/bioc/html/EDASeq.html) and any unwanted variation from the raw data is removed using RUVSeq (Risso et al., 2014) package (https://bioconductor.org/packages/release/bioc/html/RUVSeq.html ).
13. Then, edgeR (Robinson et al., 2010) package is used to obtain DE transcripts using generalized linear models. Alternatively, other packages such as DESeq2 1.30.1 (Love et al., 2014) or limma 3.46.0 (Ritchie et al., 2015) can also be used to normalize the raw counts and perform the DE analysis. The final script used in our SARS-CoV-2 protocol is given below:

perl CSBB-v3.0_MacOS.pl DifferentialExpression

 /Path_to_Local_Git/input_data/Count Data/GSE147507/Series7.txt

 3 3 10 3 UpperQuantile

14. The results from DE analysis module are written to a “temporaryfile.txt” file (tab-delimited) where the statistical p-values are adjusted for multiple-testing using the Benjamini-Hochberg procedure.

## Key resources table

**Table:** 

REAGENT or RESOURCE	SOURCE	IDENTIFIER
**Deposited data**		

Human - Calu-3 cell lines	(Blanco-Melo et al., 2020)	GEO: GSE147507
African green monkey - Vero E6 cells	(Riva et al., 2020)	GEO: GSE153940
Ad5-hACE2-sensitized mice	(Sun et al., 2020)	GEO: GSE150847
SARS-CoV-2-human virus-host PPI data	(Gordon et al., 2020)	
Human PPIs (STRING v11)	(Szklarczyk et al., 2019)	https://string-db.org/
Human lung scRNA-seq markers	(Habermann et al., 2020) (Adams et al., 2020) (Travaglini et al., 2020)	GEO: GSE135893 GEO: GSE136831 https://www.synapse.org/#!Synapse:syn21041850/
Human genome-wide phenotype associations (*PheGen*I)	(Ramos et al., 2014)	https://www.ncbi.nlm.nih.gov/gap/phegeni
Human genome-wide phenotype associations (*GWAS Catalog*)	(Buniello et al., 2019)	https://www.ebi.ac.uk/gwas/docs/file-downloads
Experimental factor ontology (EFO)	(Malone et al., 2010)	https://www.ebi.ac.uk/efo/efo.obo

**Software and algorithms**		

Proposed method	(Ghandikota et al., 2021)	https://github.com/SudhirGhandikota/COVID19_secondary_analysis
CSBB-v3.0	N/A	https://github.com/praneet1988/Computational-Suite-For-Bioinformaticians-and-Biologists
R (4.0.3)	(R Core Team, 2020)	https://www.r-project.org/
optparse (1.6.6)	(Davis, 2020)	https://cran.r-project.org/web/packages/optparse/index.html
BiocManager (1.30.10)	(Morgan, 2019)	https://cran.r-project.org/web/packages/BiocManager/index.html
biomaRt (2.46.3)	(Durinck et al., 2009)	https://bioconductor.org/packages/release/bioc/html/biomaRt.html
dplyr (1.0.6)	(Wickham et al., 2021)	https://cran.r-project.org/web/packages/dplyr/index.html
curl (4.3)	(Ooms, 2019)	https://cran.r-project.org/web/packages/curl/index.html
httr (1.4.2)	(Wickham, 2020)	https://cran.r-project.org/web/packages/httr/index.html
EDASeq (2.24.0)	(Risso et al., 2011)	https://bioconductor.org/packages/release/bioc/html/EDASeq.html
edgeR (3.32.0)	(Robinson et al., 2010)	https://bioconductor.org/packages/release/bioc/html/edgeR.html
RUVSeq (1.24.0)	(Risso et al., 2014)	https://bioconductor.org/packages/release/bioc/html/RUVSeq.html
withr (2.3.0)	(Hester et al., 2020)	https://cran.r-project.org/web/packages/withr/index.html
devtools (2.3.2)	(Wickham et al., 2020)	https://cran.r-project.org/web/packages/devtools/index.html
igraph (1.2.6)	(Csardi and Nepusz, 2006)	https://igraph.org/r/
MCL (1.0)	(Jäger, 2015)	https://cran.r-project.org/web/packages/MCL/
jsonlite (1.7.2)	(Ooms, 2014)	https://cran.r-project.org/web/packages/jsonlite/index.html
doParallel (1.0.16)	(Weston, 2020)	https://cran.r-project.org/web/packages/doParallel/index.html
stringi (1.5.3)	(Gagolewski, 2020)	http://www.gagolewski.com/software/stringi/
Python (3.6.10)	(van Rossum and Fred, 2009)	https://www.python.org/downloads/
NumPy	(Harris et al., 2020)	https://numpy.org/doc/stable/index.html
SciPy (1.4.1)	(Virtanen et al., 2020)	https://www.scipy.org/
Cytoscape visualization tool 3.8.0	(Shannon et al., 2003)	https://cytoscape.org/index.html
clusterMaker2 plugin	(Morris et al., 2011)	http://www.cgl.ucsf.edu/cytoscape/clusterMaker2/clusterMaker2.shtml
Gephi visualization tool (0.9.2)	(Bastian et al., 2009)	https://gephi.org
Enrichment analysis tool (ToppGene Suite)	(Chen et al., 2009)	https://toppgene.cchmc.org/

## Step-by-step method details

### Step 1: Obtain a consensus signature

**Timing: ∼5 min**

**Figure 1:**
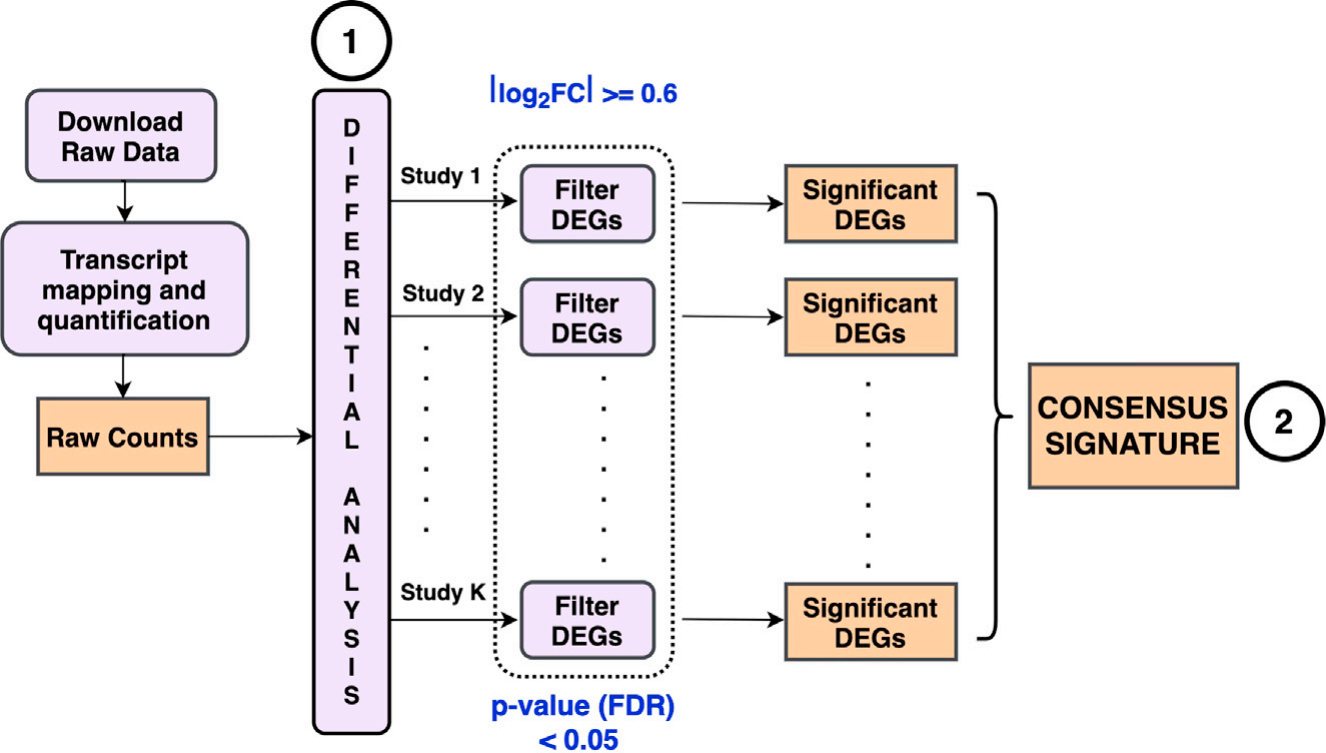
Differential expression analysis and generating a consensus transcriptomic signature First, raw transcriptomic data associated with each sample from the input studies are downloaded and processed to obtain raw transcript counts. These raw counts are then used in the differential expression step to identify the DEGs, independently within each input study. Finally, a consensus gene signature is generated from the individual gene sets (up- or down-regulated in at least 2 studies).

In this step, a consensus transcriptomic signature will be obtained by combining DEGs from each individual study. Figure 1 shows the overall workflow of DE analysis and consensus signature identification steps.

1. Using the individual DEG sets (see before you begin), the following R script can be used to filter and obtain the consensus signature:
  Rscript GetConsensus.R
   -–files “DE_results1.txt,DE_results2.txt”
   --org_assemblies GRCh38.p13,GRCm39
   --logFC 0.6 --pvalue 0.05 --k 2 --outpath 'outdir/’
  The available command-line options include:
  a. --files: comma-separated list of result files from the differential expression analysis (see before you begin), one for each study.
  b. --org_assemblies *(optional)*: comma-separated list of Ensembl assembly IDs, one for each study. Used to identify and map the human ortholog gene symbols for studies with non-human samples. Supported assembly IDs can be found at: https://uswest.ensembl.org/info/about/species.html. If not provided, all gene symbols are assumed to belong to the same organism.
  c. --logFC: log_2_fc threshold value for filtering significant DEGs (default value = 0.6)
  d. --pvalue: P-value (FDR corrected) threshold (default value = 0.05)
  e. --k: DEGs in k or more studies to be part of the consensus signature (default value = 2)
  f. --outpath: Path to the output directory where the consensus DEGs are written to.

The final conserved signature is saved in two separate files (upregulated and downregulated).

2. Firstly, filter the significant DEGs in each individual study by applying a fold change and a p-value threshold. By default, a 1.5-fold change threshold (i.e., log_2_FC ≥ 0.6 or log_2_FC ≤ −0.6) and a p-value (FDR) threshold of <0.05 is applied. Users are free to choose a different threshold value of their choice.
3. For studies involving other organisms, the corresponding human orthologs of the filtered DEGs are obtained using the biomaRt (2.46.3) R package (Durinck et al., 2009). Conversely, they can also be downloaded manually from NCBI Homologene (https://www.ncbi.nlm.nih.gov/homologene) database.
4. Next, identify consensus transcriptomic signature containing genes that are up- or down-regulated in two or more studies. Alternatively, there are several well-established meta-analysis techniques that can be used for combining data from independent studies. Fisher’s method (combined probability test) (Fisher, 1925) can be used to combine k independent statistical tests and their associated p-values to obtain one combined p-value. Similarly, Stouffer’s method and its variants (Stouffer et al., 1949; Lipták, 1958; Whitlock, 2005; Zaykin, 2011) use the inverse normal distribution to obtain a combined p-value while allowing different weights for the individual tests. But, due to the low sample counts involved in our SARS-CoV-2 specific studies, we use the above criteria for identifying the consensus DEGs.
5. Input files required to identify and reproduce the consensus signature in SARS-CoV-2 can be found in the protocol GitHub repository at COVID19_secondary_analysis/input_data/. From the three SARS-CoV-2 infection models (two *in vitro* and one *in vivo*), we observed a total of 1,467 genes (833 upregulated and 634 downregulated) that are differentially expressed in the same direction in two or more models.

### Step 2: Building consensus interactome and identifying protein modules

**Timing: ∼5–10 min**

**Figure 2:**
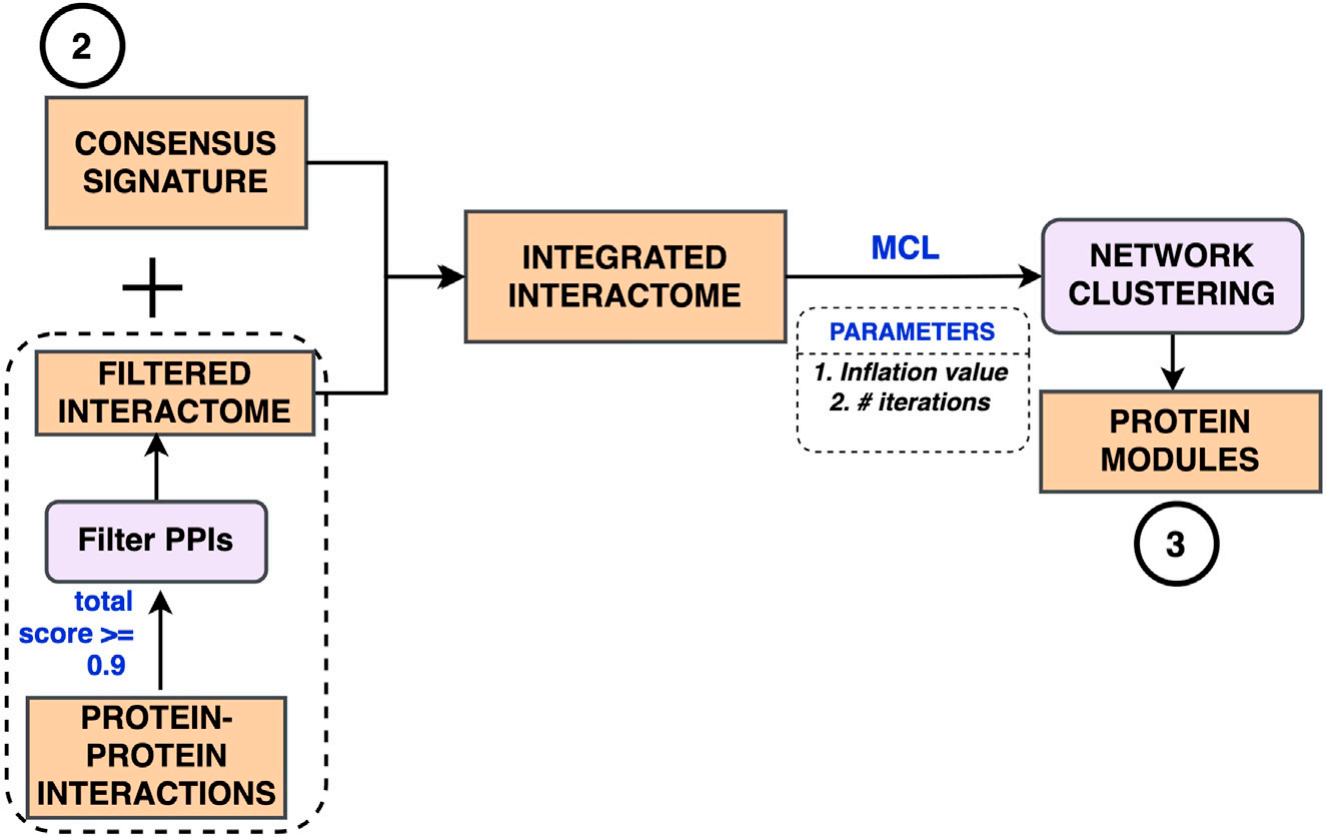
Network clustering of consensus DEG-PPI interactome to identify modules of perturbed genes The consensus transcriptomic signature is combined with curated human protein-protein interactions to build an integrated interactome. Markov clustering (MCL) is then applied on this combined interactome to identify gene/protein modules of potentially perturbed genes.

In this step a consensus interactome will be built and analyzed to identify candidate protein modules. Figure 2 indicates the different steps involved in building the integrated interactome and protein module detection.

6. The consensus transcriptomic signature from step 1 is used for building a consensus interactome of DEGs. In our SARS-CoV-2 experiments, we combine the consensus transcriptomic signature with SARS-CoV-2-proteome interacting human proteins (Gordon et al., 2020) to build a combined gene set.
7. Next, significant human PPIs (Szklarczyk et al., 2019) from within the combined gene set are identified to build an integrated DEG-PPI network. While there are other curated PPI resources like BioGRID (4.4.199) (Oughtred et al., 2021), HuRI (Luck et al., 2020) and PINA v1.0 (Wu et al., 2009) available in the literature, we used STRING (v11) because of its extensive coverage and the ability to filter the interactions based on the source information (experimental, text-mining etc.,)
  a. This can be achieved by manually uploading the list of proteins to https://string-db.org/ and exporting all the PPIs using the *Exports* option (TSV) from the result screen. The resultant output file contains all edges among the uploaded proteins.
  b. However, the web-interface is not designed to handle more than 2000 proteins as input, in which case it is better to download the full PPI data and filter the interactions manually. We provide a script (described below) as part of this protocol which takes in the full set of PPIs and applies a filtering criterion of choice.
  c. The STRING-based human PPI network data is known to suffer from noise and incompleteness. To overcome these issues, only likely interactions (true positives) are used in this protocol by filtering the edges based on combined_score ≥ 0.9 (column 13) or experimental_score ≥ 0.7 (column 10). Users can select a different threshold to filter the final set of interactions.
  d. Our GitHub repository includes a filtered set of PPIs (https://github.com/SudhirGhandikota/COVID19_secondary_analysis/tree/main/input_data/other%20data/filtered_PPI.txt) from STRING (v11) that we used in our SARS-CoV-2-specific analysis.

8. Eventually, network modules from this joint interactome are identified using the Markov clustering (MCL) algorithm (Enright et al., 2002).
  a. To do this, import the filtered interactome into the Cytoscape (Shannon et al., 2003) visualization tool (3.8.0) and analyze it using MCL algorithm in the clusterMaker2 (Morris et al., 2011) plugin. MCL works by simulating multiple random walks within the input network combined with alternative steps of inflation and expansion (Saelens et al., 2018).
  b. The inflation factor parameter of the MCL algorithm (https://micans.org/mcl/man/mcl.html) determines the tightness of the identified clusters. Increasing its value leads to higher granularity (smaller clusters) and vice-versa. The default inflation value of 2.5 is used in this protocol. Results from clustering analysis, including the membership information, can be downloaded directly from the Cytoscape tool.
  c. Optionally, we also provide an R Script which implements the MCL analysis step using the following syntax:
    Rscript MCL_Clustering.R
     --deg_file ‘../input_data/SARS-CoV2_DEGS/DEGs+PPI.txt’
     --PPI_file ‘../input_data/other\ data/filtered_PPI.txt’
     --filter 'combined_score >= 900'
     --inflation_value 2.5 --max_iter 100
     --outpath ‘../input_data/other\ data/’
    The command-line parameters to the script include:
    i. --deg_file: A file containing the consensus DEGs and the virus-host interactome, if used (one gene per each line).
    ii. --PPI_file: (Optional) A tab-delimited file containing the set of human PPIs. The latest version of STRING human PPIs can be downloaded from the following link https://string-db.org/cgi/download?species_text=Homo+sapiens. Conversely, interactions from other sources such as HuRI or BioGRID can also be provided (minus the --filter parameter). By default, the script uses the filtered STRING PPIs used in the protocol, which could be found at https://github.com/SudhirGhandikota/COVID19_secondary_analysis/tree/main/input_data/other%20data on the project GitHub page.
    iii. --filter: A condition to filter the PPI links (STRING only) prior to running the clustering algorithm. In our protocol, we only retained the edges with a *combined_score ≥ 0.9* or *experimental_score ≥ 0.7.*
    iv. --inflation_value: Inflation parameter in MCL algorithm. It determines granularity of the identified clusters. High inflation values would result in increased granularity (smaller clusters) and vice-versa (default value = 2.5).
    v. --max_iter: Maximum number of iterations for the MCL algorithm (default value = 100).
    vi. --outpath: Path to the output directory where the file containing the final MCL cluster memberships are written to.
  d. While there are several other clustering algorithms to choose from, MCL is ideal for identifying dense gene modules (Ghiassian et al., 2015) with more intramodular (within the same module) interactions than intermodular (other modules). These modules have been found to reveal the functional effects from within the gene expression networks (Saelens et al., 2018). Additionally, for unweighted PPI networks, the MCL procedure has been found to be robust and tolerant to any input noise (Vlasblom and Wodak, 2009; Brohee and van Helden, 2006).

### Step 3: Identify high-level feature complexes by constructing an enriched feature network

**Timing: 35–40 min**

**Figure 3:**
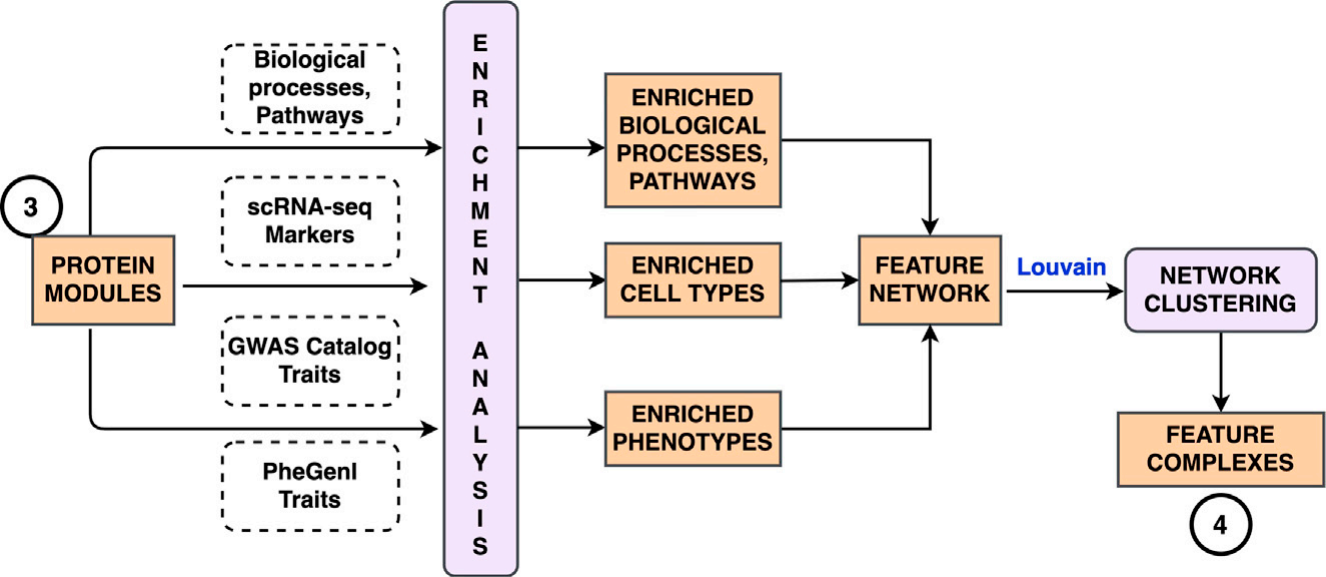
Joint analysis of enriched annotations from candidate gene modules to obtain feature complexes Candidate gene modules (>= 5 genes) are first tested for enrichments of functional features (biological processes and pathways), scRNA-seq markers and phenotype associations (from GWAS Catalog and PheGenI). An enriched feature network is then constructed using the enriched terms among all the candidate modules. Finally, a community detection methodology (Louvain method) is applied to identify functional complexes of enriched features.

As a precursor to this step, candidate modules from step 2 are filtered based on the number of genes within them and functional enrichment analyses is conducted to identify the enriched cell types, phenotypic traits, and functional terms. Using a selection criterion of >= 5 genes, a total of 35 candidate modules were identified from the integrated SARS-CoV-2 interactome map. The overall workflow of this step can be seen in Figure 3.

9. Compute functional enrichments of Gene Ontology-Biological processes, Reactome pathways and mouse phenotypes among the candidate gene modules coming out of the MCL algorithm. We used ToppCluster application (Kaimal et al., 2010) (https://toppcluster.cchmc.org) of the ToppGene suite (Chen et al., 2009) in this protocol to compute the enrichments within each cluster.
10. Top enriched terms from each category (for each module) are used towards building the feature network. While there are several enrichment analyses tools available (Chen et al., 2013; Eden et al., 2009; Ghandikota et al., 2018; Jiao et al., 2012; Mi et al., 2018), ToppCluster allows us to simultaneously identify enriched terms from multiple gene lists representing the different clusters. Similarly, Metascape (Zhou et al., 2019) is another useful tool that can be used to analyze multiple clusters and generate functional feature complexes from them. Further, researchers can select different sets of annotation categories and functional terms depending on the research questions.

11. Next, evaluate the modules for enrichments of single-cell marker gene lists. For filtered SARS-CoV-2 gene modules we computed enrichment analysis against three different lung scRNA-seq studies (Adams et al., 2020; Habermann et al., 2020; Travaglini et al., 2020). Using marker gene lists published in each of these studies, we identified all the enriched cell types (FDR p-value ≤ 0.05) within each candidate module. The RScript ***Marker_enrichments.R***, present in the project GitHub page, can be used to perform the cell-type marker enrichments with the syntax shown below:
  Rscript Marker_enrichments.R
   --marker_file ../input_data/Lung_Markers/lung_markers_test.txt
   --p_value 0.05 --logFC 0.5
   --cluster_file ../input_data/SARS-CoV-2-Cons_MCL_Clusters.txt
   --outpath outdir
  a. --marker_file: Text file containing the cell type marker genes. This file should contain 4 mandatory columns corresponding to the cell type (“cell”), gene marker (“gene”), fold change (“logFC”) and the adjusted p-value (“pval_adj”).
  b. --p_value and --logFC: The p-value (multiple-testing adjusted) and fold change thresholds to filter the marker genes.
  c. --cluster_file:A two column, tab-delimited file containing genes (column 1) and their corresponding MCL cluster memberships (column 2)
  d. --min_genes: Minimum number of genes needed to be in a candidate cluster (default value = 5).
  e. --outpath: Path to the output directory where the marker enrichment results need to be stored.

12. Thereafter, test the filtered gene modules for enrichments of phenotypic traits using compiled genotype-phenotype associations from the NCBI PheGenI (Ramos et al., 2014) and the NHGRI-EBI GWAS Catalog (Buniello et al., 2019) databases.
  a. In case of GWAS Catalog associations, child terms for each trait are parsed from the experimental factor ontology (EFO) hierarchy (Malone et al., 2010) and used in the enrichment step while the PheGenI traits are used as it is.
  b. We provide a python script ***GWAS_enrichments.py*** which first parses the EFO tree to obtain the child terms for each GWAS Catalog trait and then computes their enrichments among the SARS-CoV-2-specific candidate modules.
    python GWAS_enrichments.py
    --obo_file ../input_data/other\ data/efo.obo.txt
    --cluster_file ../input_data/SARS-CoV-2-Cons_MCL_Clusters.txt
    --assoc_file ../input_data/other\ data/gwas_catalog_v1.0.2-
    associations_e100_r2020-07-14.tsv --outpath outdir
    The EFO open biomedical ontology (OBO) file (https://www.ebi.ac.uk/efo/efo.obo) and a tab separated file containing the GWAS Catalog associations (https://www.ebi.ac.uk/gwas/docs/file-downloads) used in our work are also included in the GitHub repository.
  c. Similarly, we also include an R Script ***PheGenI_enrichments.R*** to compute enrichments among the PheGenI traits using the below syntax:
    Rscript PheGenI_enrichments.R
    --assoc_file ../input_data/other\data/PheGenI_Associations.txt
    --cluster_file ../input_data/SARS-CoV-2-Cons_MCL_Clusters.txt
    --remove_intergenic --p_value 0.00001 --outpath outdir
  d. Both these scripts support the following set of command-line parameters:
    i. --obo_file (for GWAS script only): Path to the EFO OBO file (.txt) which can be found at https://www.ebi.ac.uk/efo/efo.obo.
    ii. --assoc_file: A Tab-delimited file containing phenotype-genotype associations from NCBI PheGenI (https://www.ncbi.nlm.nih.gov/gap/phegeni) or GWAS Catalog associations (https://www.ebi.ac.uk/gwas/docs/file-downloads).
    iii. --cluster_file: A two column, tab-delimited file containing genes (column 1) and their corresponding MCL cluster memberships (column 2).
    iv. --min_genes: Minimum number of genes needed to be in a candidate cluster (default value = 5).
    v. --p_value (for PheGenI associations only): To specify a p-value threshold for filtering associations (default value = 1e-05).
    vi. --remove_intergenic: A Boolean flag to indicate the removal of intergenic associations. In our SARS-CoV-2-specific protocol, all intergenic associations from both PheGenI and GWAS Catalog were ignored.
    vii. --outpath: Path to the output directory where the enrichment results need to be stored.

13. Finally, construct a term-term network using the enriched features (pathways, biological processes, cell types, phenotypic traits) from the candidate gene modules. Only a subset of enriched functional terms (top ten GO-BP, pathways and mouse phenotypes based on negative log p-values) are considered to reduce the complexity and manage the density of the final feature network. This filtering step however is optional, especially if the candidate cluster counts and/or the enriched terms are smaller in number. Two feature nodes are connected by an edge if they share one or more of the module candidates.
14. This functional network is visualized and analyzed using Gephi (Bastian et al., 2009), an open-source visualization tool (https://gephi.org). The input to the Gephi tool (version 0.9.2) is a tab-delimited file containing the source and destination enriched terms. Using the Louvain clustering (Blondel et al., 2008) method, community membership modules are estimated from the enriched feature network.
15. These functional modules or higher-order functional complexes are hypothesized to be involved in similar biological mechanisms underlying a disease pathophysiology. Louvain clustering algorithm is selected for this step because it is computationally efficient and suitable for large, dense, and modular networks, commonly seen in feature networks such as this. Gephi provides a resolution parameter that maintains the balance between the module count and individual cluster tightness with lower values leading to smaller, more tightly connected clusters and vice-versa. With a resolution set to 0.25, we found 31 communities of highly connected functional terms from the SARS-CoV-2-specific feature network.
16. The identified gene modules (step 2) and the higher-order feature-level complexes from this meta-analysis approach can be manually analyzed or curated for either knowledge extraction or to formulate testable hypotheses to understand or characterize a disease or phenotype.

## Expected outcomes

### Consensus gene signature

One of the first outcomes from our protocol include the consensus gene signature from all the input transcriptomic studies (step 1). In total, we found 1467 genes (833 upregulated and 634 downregulated) that are differentially expressed in at least two out of the three SARS-CoV-2 infection model systems. Among these, 147 genes are either upregulated (106 genes) or downregulated (41 genes) in all the three studies, representing a potential “core” transcriptomic signature. Figure 2 in (Ghandikota et al., 2021) indicates the transcriptomic concordance among the input models in addition to the different enriched biological processes and pathways in the core signature.

### Disease-targeted human protein modules

Another outcome from our protocol is the list of protein modules identified from an integrated interactome consisting of consensus DEGs (step 2). From the three distinct SARS-COV-2 infection models, we identified 35 candidate modules (at least 5 or more genes) from a combined network of 1,467 consensus DEGs and SARS-CoV-2-human virus-host interactome (336 genes). Among these, 29 modules have at least one gene encoding SARS-CoV-2-host interactant protein. Figure 3C in (Ghandikota et al., 2021) shows example gene modules from the consensus DEG and SARS-CoV-2-host integrated interactome identified using the current protocol. These modules potentially represent the different perturbed states within a disease and are further characterized by computing enrichments of different functional terms, cell types, and phenotypic traits.

### Higher-order multifeature complexes

The final outcome from our protocol includes functional complexes identified through a meta-analysis of enriched features from candidate protein modules (step 3). These higher-order feature networks could help in identifying interpretable biological mechanisms underlying a disease pathophysiology. The semantic concordance observed among the subunits (e.g., pathways, cell types, biological processes, and phenotypes) of these functional complexes suggests a common biological goal. In total, 31 communities of strongly connected functional features were estimated from a dense feature network (1,198 nodes and 31,065 edges) based on 35 selected gene modules in SARS-CoV-2 infection. Figure 5 in (Ghandikota et al., 2021) represents a network visualization from the meta-analysis of different functional annotations and features from SARS-CoV-2 candidate modules.

### Intermediate and final results

“Consensus_up.txt” and “Consensus_down.txt” – Text files containing the lists of genes (one gene per line) corresponding to upregulated and downregulated consensus signatures, respectively. By default, these files consist of genes differentially expressed (log_2_FC ≥ 0.6 or log_2_FC ≤ −0.6; p-value (FDR) <0.05) in 2 or more input studies.

“MCL_Clusters.txt” – A tab-delimited text file containing the results from the MCL clustering step. Each row contains a gene and the corresponding cluster membership i.e., the cluster ID that it belongs to. The result file might also include genes which do not belong to any of the identified clusters, in which case, the cluster membership value will be empty.

“module_PheGenI_enrichments.txt” and “module_GWAS_enrichments.txt” – Output files having results from the enrichment tests computed between all the candidate modules and phenotypic traits from both PheGenI and GWAS Catalog respectively. Apart from the enrichment test statistics (overlap size, both adjusted and raw p-values, negative log p-value), these files also include a column with comma-separated list of genes that are in common between the given module and a phenotypic trait. In case of the GWAS Catalog traits, gene associations of the corresponding child traits (from EFO hierarchy) are also used in the enrichment tests. The counts of mapped child traits are also specified in the output file.

“module_marker_enrichments.txt” – A tab-delimited output file containing cell type marker genes enriched among the candidate modules. The overlapping marker genes are included in a separate column, as a comma-separated list.

## Limitations

The outcomes (consensus DEGs, protein modules and functional complexes) in our protocol are dependent on certain assumptions and choices made during the analysis steps. Firstly, the composition of the protein modules is dependent on the consensus transcriptomic signature and the PPIs identified among them. Any noise or heterogeneity in the model systems or the transcriptomic data can impact the transcriptomic concordance between the different model systems and the consensus signature (step 1) used to build the interactome. Additionally, smaller sample sizes in the individual disease models can impact the final set of consensus DEGs. Similarly, PPI from STRING, as is the case with most large-scale compiled annotation resources, may be prone to noise and incompleteness in the data. Although used to reduce the noise, selection of a high STRING cutoff score can impact the compositions of the gene modules from the DEG and PPI integrated interactome used in step 2.

The final module composition (gene/protein assignment to the modules) also depends on the choice of clustering algorithms and the parameter values used to generate them. For instance, increasing the inflation factor parameter value of the MCL algorithm would lead to smaller-sized clusters or modules and vice-versa (step 2). Although, we used the default inflation factor parameter value (=2.5) in the clustering step, it would be prudent to review the results using different inflation factor values. Similarly, modifying the resolution parameter of Louvain clustering can affect the composition of the final functional complexes identified in our protocol (step 3).

Finally, the functional complexes are based on the set of functional terms or annotations used in the enrichment step, which in turn, is dependent on the disease or phenotype being studied. Any errors or redundancy within the annotation sources (e.g., biological processes, single cell markers, phenotype associations, etc.) can affect the composition of the functional complexes identified.

## Troubleshooting

### Problem 1

Error message “*Error in getopt(spec = spec, opt = args) : long flag XXXX is invalid*” while using any of the R Scripts.

### Potential solution

This error message commonly occurs when a wrong command-line parameter option flag (e.g., *--files*) is used to run an R script. The specified option flag needs to be fixed before proceeding with the execution of the corresponding R Script.

### Problem 2

Error message “*Number of species assemblies do not match the number of input files provided*” while running the script “*GetConsensus.R*”.

### Potential solution

This error occurs when the number of Ensembl assembly IDs (comma-separated), provided explicitly using the *--org_assemblies* option, does not match the study count (comma-separated list of files using the *–files* option) in protocol step 1. In this case, the species Ensembl assembly IDs should be explicitly provided for each input study. Otherwise, the option can be skipped altogether if all the studies involve human samples.

### Problem 3

Warning message “*Unable to recognize the provided Ensembl assembly*” while running the script “*GetConsensus.R*”.

### Potential solution

This message indicates that the provided assembly ID(s) (in step 1) is unrecognizable by Ensembl (Durinck et al., 2009). In this case, the original gene inputs will be directly used (skipping the ortholog mapping step). The list of valid species assembly IDs can be found at the following URL: https://uswest.ensembl.org/info/about/species.html.

### Problem 4

Error message “*Error in mcl_results$Cluster : $ operator is invalid for atomic vectors*” while implementing the MCL step using the RScript “*MCL_Clustering.R*”.

### Potential solution

This message potentially occurs if the number of iterations of the MCL algorithm (step 2) were not sufficient to identify the clusters within the combined interactome. Increasing the maximum number of iterations using the *“--max_iter*" command-line parameter could help prevent this error message.

### Problem 5

Error message “*Error: Small inflation coefficient prevents that an equilibrium state matrix is reached within XXX iterations*” while using the R Script “*MCL_Clustering.R*”.

### Potential solution

This message indicates that the given combination of inflation coefficient parameter and the number of MCL iterations are not sufficient for the modularity objective to converge during Step 2. This could be prevented by using a higher inflation parameter value using the *--inflation_value* command line option or by increasing the maximum of iterations using the *--max_iter* command-line parameter.

## Resource availability

### Lead contact

Further information and requests should be directed to and will be fulfilled by the lead contact, Anil G. Jegga (anil.jegga@cchmc.org).

### Materials availability

This study did not generate any unique reagents.

## Data Availability

The scripts to implement the proposed protocol are accessible publicly at https://github.com/SudhirGhandikota/COVID19_secondary_analysis. It also includes a Dockerfile that can be used to build and run a docker container which has all the tools, packages, and their dependencies installed. The repository also contains the results from our analysis of SARS-CoV-2 infection models.
